# Initiation, Elongation, and Realignment during Influenza Virus mRNA Synthesis

**DOI:** 10.1128/JVI.01775-17

**Published:** 2018-01-17

**Authors:** Aartjan J. W. te Velthuis, Judith Oymans

**Affiliations:** a Sir William Dunn School of Pathology, University of Oxford, Oxford, United Kingdom; b University of Cambridge, Department of Pathology, Division of Virology, Addenbrooke's Hospital, Cambridge, United Kingdom; St. Jude Children's Research Hospital

**Keywords:** influenza A virus, RNA-dependent RNA polymerase, replication, transcription, priming loop, realignment, viral transcription

## Abstract

The RNA-dependent RNA polymerase (RdRp) of the influenza A virus replicates and transcribes the viral genome segments in the nucleus of the host cell. To transcribe these viral genome segments, the RdRp “snatches” capped RNA oligonucleotides from nascent host cell mRNAs and aligns these primers to the ultimate or penultimate nucleotide of the segments for the initiation of viral mRNA synthesis. It has been proposed that this initiation process is not processive and that the RdRp uses a prime-realign mechanism during transcription. Here we provide *in vitro* evidence for the existence of this transcriptional prime-realign mechanism but show that it functions efficiently only for primers that are short or cannot stably base pair with the template. In addition, we demonstrate that transcriptional elongation is dependent on the priming loop of the PB1 subunit of the RdRp. We propose that the prime-realign mechanism may be used to rescue abortive transcription initiation events or cope with sequence variation among primers. Overall, these observations advance our mechanistic understanding of how influenza A virus initiates transcription correctly and efficiently.

**IMPORTANCE** Influenza A virus causes severe disease in humans and is considered a major global health threat. The virus replicates and transcribes its genome by using an enzyme called the RNA polymerase. To ensure that the genome is amplified faithfully and abundant viral mRNAs are made for viral protein synthesis, the viral RNA polymerase must transcribe the viral genome efficiently. In this report, we characterize a structure inside the polymerase that contributes to the efficiency of viral mRNA synthesis.

## INTRODUCTION

**FIG 1:**
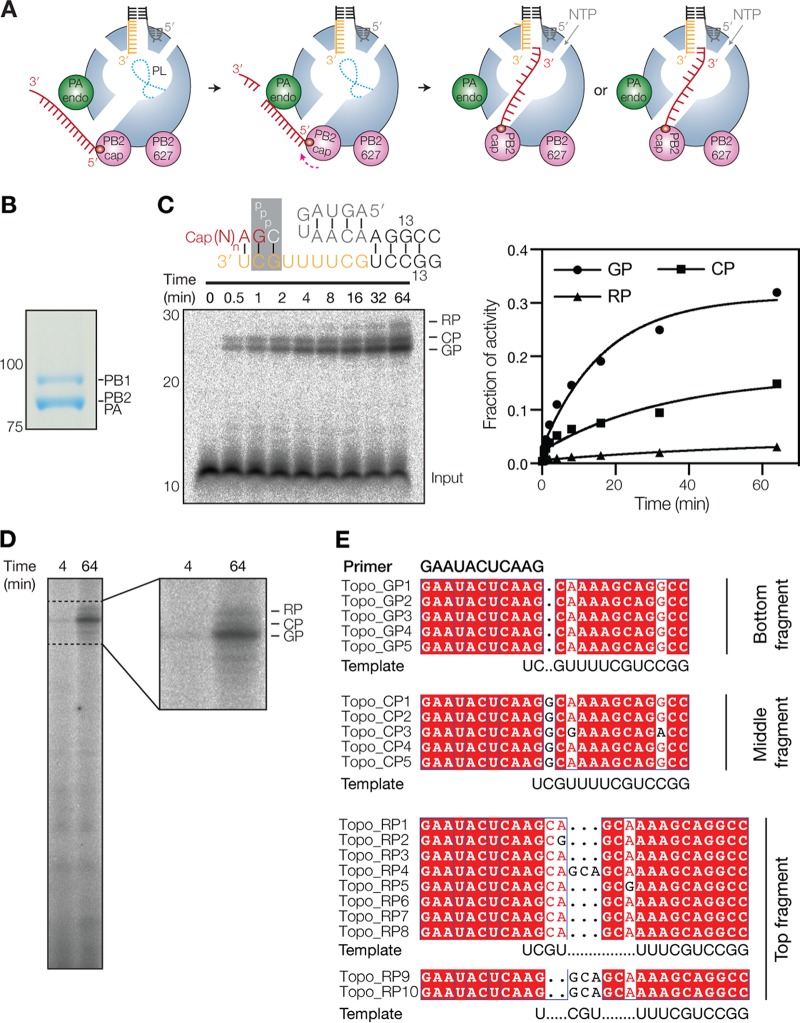
Transcription initiation by the influenza virus RdRp produces three RNA species. (A) Schematic of cap cleavage and transcription initiation with a capped 11-nucleotide-long primer. The duplex of the viral promoter is shaded black, the 3′ end is in orange, and the 5′ end is in gray. The capped primer is shaded red, and the priming loop (PL) is drawn as a dotted line. Alignment of the capped primer to 1U or 2C is also depicted. (B) SDS-PAGE analysis followed by Coomassie staining of purified IAV RdRp. (C) Extension of a radiolabeled, capped, 11-nucleotide-long primer in the presence of four NTPs. The products produced from positions 2C (CP) and 3G (GP) as well as the realignment product (RP) are indicated. The promoter schematic shows the primary initiation site on the wild-type IAV promoter, with colors as described above for panel A. The graph shows the accumulation of the GP, CP, and RP signals as a fraction of the total transcriptional activity. Lines represent fits to an exponential decay of data from one representative experiment. (D) Transcription reaction using β-globin mRNA as the primer donor. (E) Alignment of IAV transcription products present in the GP, CP, or RP band-containing gel fragments as identified by TOPO cloning and Sanger sequencing.

RNA viruses use an RNA-dependent RNA polymerase (RdRp) to replicate and transcribe their viral RNA (vRNA) genome. One of the best-studied negative-strand RNA viruses is the influenza A virus (IAV). The IAV genome is replicated and transcribed in the nucleus of the host cell by the IAV RdRp, an enzyme that consists of the viral proteins PB2, PB1, and PA (1, 2). The N-terminal one-third of PB2, the PB1 subunit, and the C-terminal two-thirds of PA form the conserved core of the RdRp (3–5), while the remaining portions of PB2 and PA form flexible domains that have cap-binding and endonuclease activities, respectively (Fig. 1A). Another key functional structure in the RdRp is a conserved PB1 β-hairpin called the priming loop, which resides downstream of the active site of the IAV RdRp and is important for viral replication initiation (4, 6, 7). The promoter for the IAV RdRp is bound by the conserved core of the RdRp and is known to consist of the partially complementary 3′ and 5′ ends of the vRNA genome segments (Fig. 1A) (3, 4).

Unlike IAV replication, in which the RdRp initiates *de novo* (6), the IAV RdRp uses a primer-dependent process for viral transcription initiation. To produce this primer, the IAV RdRp must first bind to the C-terminal domain of an actively transcribing, serine 5-phosphorylated RNA polymerase II (Pol II) complex in the nucleus of an infected cell (8, 9). Subsequent binding and cleavage of nascent Pol II transcripts produce 8- to 14-nucleotide (nt)-long capped RNAs (10) (Fig. 1A) that the IAV RdRp can hybridize as primers to the 3′ terminus of the vRNA template (Fig. 1A). The PA endonuclease domain has a preference for mRNA cleavage 3′ of G residues *in vitro* (11, 12), which creates primers that can be hybridized with the penultimate C residue (2C) of the 3′ terminus of the vRNA template (Fig. 1A). This match between cleavage preference and template sequence is also reflected in a recent crystal structure of the influenza B virus RdRp that is bound to a vRNA and capped primer (13), because it showed that the 3′ terminus of the vRNA can “overshoot” the active site by 1 nt without duplex unwinding. Hence, ostensibly by default, the RdRp positions 2C of the vRNA in the −1 position of the active site (13), which is ideal for transcription initiation with primers ending in 3′ G from 3G of the template. However, in viral infections and other *in vitro* studies, capped RNA primers with other 3′-terminal bases are also frequently produced and used (14–18), and current evidence suggests that these primers are extended from 2C instead of 3G (Fig. 1A).

After transcription initiation, the RdRp extends the primer in a template-dependent fashion. However, IAV mRNAs isolated from infected cells often contain 3-nt repeats (GCA or GCG, depending on the segment) that are complementary to the second, third, and fourth nucleotides of the template (14, 15). This observation implies that RdRp processivity is limited over the first 4 bases of the vRNA. It has been proposed that the 3-nt repeats are introduced by the IAV RdRp through a realignment mechanism (14, 15, 18), but direct evidence for this process is currently lacking. Moreover, it is also not known whether there is a link between the generation of capped primers in the host nucleus, the ability of the RdRp to hybridize these primers efficiently to the 3′ terminus of the vRNA template, and the generation of these 3-nt duplications.

In this study, we use a combination of structure-guided mutagenesis and *in vitro* polymerase activity assays to provide *in vitro* evidence for the existence of low-processive transcription elongation events that result in a duplication of the first 3 nucleotides of the vRNA 3′ terminus. Moreover, we show that the synthesis of these duplications is dependent on the sequence of the capped primer, the sequence of the template, and the interaction of the body of the RNA primer with the body of the priming loop. These observations thus provide mechanistic insight into IAV RNA synthesis and redefine the function of the priming loop as a platform for both efficient replication (6, 7) and transcription.

(This article was submitted to an online preprint archive [19].)

## RESULTS

### The initiation of IAV transcription produces multiple products.

IAV transcription uses a capped RNA primer that is snatched from host cell mRNAs and subsequently hybridized to the 3′ 1U and/or 2C of the vRNA promoter for extension from 3′ 2C or 3G, respectively (Fig. 1A). Although it is currently assumed that this process is dependent largely on Watson-Crick base pairing between the primer and the template, transcription initiation without Watson-Crick base pairing has been observed (14–18). To study IAV transcription initiation in detail, we expressed the PB1, PB2, and PA subunits of the influenza A/Northern Territories/60/1968 (H3N2) virus in insect cells (9) and purified the recombinant enzyme using a tandem affinity purification (TAP) tag on PB2, as described previously (5). The purified enzyme was analyzed by SDS-PAGE for purity (Fig. 1B). Next, we set up reaction mixtures containing the IAV RdRp with an 11-nt radiolabeled capped RNA ending in 3′ AG (AG primer) and monitored the extension of the primer in time (Fig. 1C). PAGE analysis showed that the extension of the primer resulted in one major product and two slower-migrating minor products (Fig. 1C). The slowest-migrating product was produced ∼10 times less efficiently than the major product (Fig. 1C). Transcription products similar to the ones described above were previously observed in assays in which IAV RdRp purified from mammalian cells was used to extend an 11-nt-long capped primer (6, 7) or a β-globin mRNA-derived primer (20–24) (Fig. 1D), suggesting that these three RNA species are typical IAV transcription products.

To characterize the three products in more detail, we cut the part of the gel containing the three bands into three fragments and extracted the RNA contained in the three gel fragments. Subsequent Sanger sequencing analysis revealed that the gel fragment with the bottom band contained an RNA species that had initiated at 3′ 3G of the template (Fig. 1E), whereas the fragment with the second band contained a product that had been produced after initiation at 3′ 2C of the template (Fig. 1E). The third fragment, which included the third band and a small part of the gel above this band, was found to contain three RNA species (Fig. 1E), all of which had at least one duplication of residues 2 to 4 of the template, similar to the RNA species observed in IAV infections (14, 15, 18). Moreover, the RNA species contained products that had initiated from both 3′ 2C and 3′ 3G, implying that the duplication of residues 2 to 4 during transcription elongation occurs independently of the site of transcription initiation. Here we refer to the identified RNA species as the 3G-initiated product (GP), the 2C-initiated product (CP), and the 3-nucleotide repeat products (RPs) for simplicity.

### Realignment during transcription is dependent on 3′ 1U and 4U of the vRNA template.

**FIG 2:**
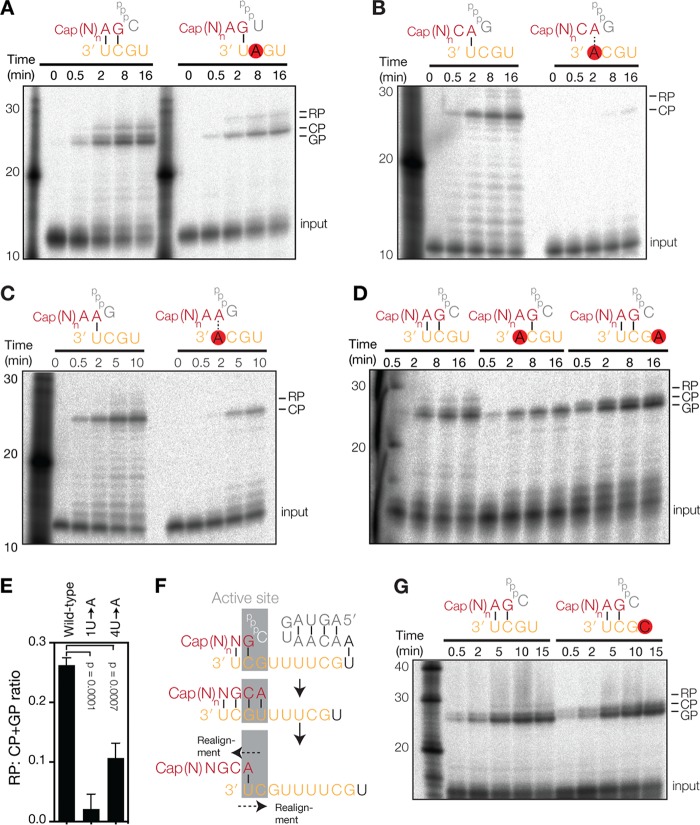
Realignment during IAV transcription involves 4U and 1U of the template strand. (A) Extension of radiolabeled, capped, 11-nucleotide-long RNA in the presence of unlabeled NTPs and IAV RdRp. The schematic shows the primary initiation site on the wild-type or mutant 2C→A 3′ promoter strand. (B) Extension of a radiolabeled, capped, 11-nucleotide-long RNA primer ending in 3′ CA on the wild-type vRNA promoter or the 3′ 1U→A mutant promoter. (C) Extension of a radiolabeled, capped, 11-nucleotide-long RNA primer ending in 3′ AA on the wild-type vRNA promoter or the 3′ 1U→A mutant promoter. (D) Extension of a radiolabeled, capped, 11-nucleotide-long RNA primer ending in 3′ AG on the wild-type, 3′ 1U→A, or 3′ 4U→A vRNA promoter. (E) Quantitation of data in panel D. The graph shows mean ratios of RP to GP plus CP from 3 independent assays. Error bars indicate standard deviations. The *P* values were determined by using an unpaired *t* test. (F) Model of realignment after initiation from 3G. Colors are as described in the legend of Fig. 1A. (G) Extension of a radiolabeled, capped, 11-nucleotide-long RNA primer ending in 3′ AG on the wild-type vRNA promoter or a 3′ 4U→C mutant promoter.

To verify that the GP species had indeed been synthesized after initiation at 3′ 3G in our assay, we first mutated 3′ 2C of the vRNA promoter to A (2C→A) in order to disrupt G-C base pairing between the AG primer and template. As shown in Fig. 2A, this mutation resulted in a loss of the GP signal and a concomitant increase in the CP signal (Fig. 2A), likely due to an increase in G-U base pairing between the AG primer and template. (Note that the CP band generated on the mutant template migrates between the GP and CP bands that were produced on the wild-type template. This difference is due to a G-to-U change, which reduces the molecular weight of the CP band and increases its mobility in 20% PAGE gels.) For the second control, we replaced the AG primer with a primer ending in 3′ CA and incubated it with the wild-type promoter, nucleoside triphosphates (NTPs), and the RdRp. As shown in Fig. 2B, this reaction yielded a similar level of the CP product, confirming that GP synthesis is dependent on primer base pairing with 3′ 2C and initiation at 3′ 3G. A similar result was obtained when we used a primer ending in 3′ AA (Fig. 2C). To fully verify that CP synthesis is dependent on base pairing with 1U, we next mutated 3′ 1U of the vRNA promoter to A (1U→A) and incubated this promoter with the AG primer and the IAV RdRp. We found that in this reaction, both the CP as well as the RP levels were significantly reduced (Fig. 2D and E), which confirms that CP synthesis is dependent on primer base pairing with 3′ 1U. Interestingly, when we incubated the CA or the AA primer with the 1U→A template, we found that transcription initiation was severely impaired, suggesting that A-A base pairs do not support efficient transcription initiation.

In the above-described experiments, RP formation was independent of 3′ 2C but dependent on 3′ 1U (Fig. 2D). This is in line with the idea that the RP species are formed through a realignment event between positions 4U and 1U (Fig. 2F). To confirm this, we mutated 4U to A (4U→A) in the 3′ strand of the vRNA promoter and found that this mutation supported CP and GP formation but greatly reduced RP synthesis (Fig. 2D). Indeed, quantitation of the product levels showed that the ratio between the RP signal and the sum of the CP and GP (CP+GP) signals was significantly diminished (Fig. 2E). A similar result was obtained when we mutated 4U to C (4U→C) (Fig. 2G), a sequence variant that downregulates the transcription of segments 1, 2, and 3 of the IAV genome (25). Overall, these findings imply that Watson-Crick base pairing between the primer and the template plays an important role during transcription initiation and that Watson-Crick base pairing between the extended primer and 3′ 1U is crucial for RP formation when transcription elongation is not processive.

### Duplex stability and ssRNA primer length affect transcription processivity.

**FIG 3:**
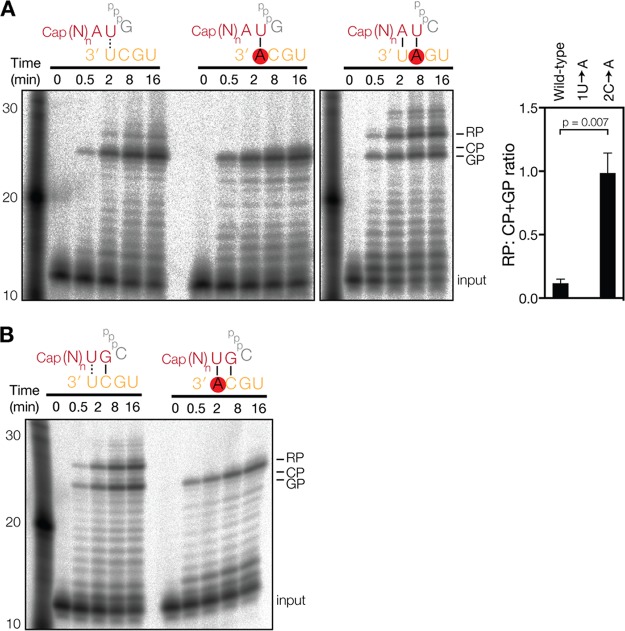
Transcription elongation is affected by the primer sequence. (A) Extension of radiolabeled, capped, 11-nucleotide-long RNA with a 3′ AU sequence in the presence of four NTPs. The graph shows mean ratios of RP to GP plus CP from 3 independent assays. Error bars indicate standard deviations. The *P* values were determined by using an unpaired *t* test. (B) Extension of a radiolabeled, capped, 11-nucleotide-long primer with a 3′ UG sequence on the wild-type vRNA promoter or the 3′ 1U→A mutant promoter.

The above-described results imply that transcription initiation relies on base pairing when the IAV RdRp uses primers ending in AG or CA. However, the IAV RdRp can also use primers that are not complementary to the template (15, 16, 18). To investigate how such noncomplementary primers affect transcription initiation and elongation processivity, we first replaced the terminal G base of the AG primer with U (i.e., creating a primer ending in 3′ AU [AU primer]). On the wild-type vRNA promoter, this AU primer was efficiently extended into a major CP signal and minor GP and RP bands (Fig. 3A). To confirm that the AU primer was indeed primarily extended from 3′ 2C, we replaced the wild-type promoter with the 1U→A mutant promoter, because this would enforce U-A base pairing with 3′ A of the mutant promoter and increase the CP/GP signal ratio. Indeed, the control reaction yielded a similar CP signal and a weak GP band (Fig. 3A). The RP signal was severely reduced in this reaction, due to the 3′ 1U-to-A mutation in the template, which prevented realignment during nonprocessive elongation. Thus, we find that U-U base pairs support transcription initiation, while A-A base pairs do not (Fig. 2B and C).

To investigate if we could make the AU primer initiate from G3, we next used the 2C→A mutant promoter in the reaction (Fig. 3A) and found that A-U base pairing at positions 1 and 2 of the primer-template duplex supports GP formation. Surprisingly, this altered initiation duplex also upregulated realignment (Fig. 3A) relative to the wild-type template and the 3′ AG primer reaction (Fig. 1C). In addition, we observed multiple RP bands of higher molecular weights (Fig. 3A), in line with the multiple RP sequences identified in Fig. 1E and elsewhere (15). Together, these results suggest that the duplex that is formed between the 2C→A mutant promoter and the AU primer is less stable and that this reduces the processivity of the IAV RdRp. However, the difference in stabilities among the various primers and promoters tested is minimal, particularly when we take into account their partial extension products. For instance, the duplex formed during extension of the AU primer on the wild-type promoter is $\frac{UGCA}{UCGU}$, whereas the duplex is $\frac{AUCA}{UAGU}$ when the AU primer is extended on the 2C→A mutant promoter. We therefore reasoned that the stability of the duplex could not solely explain the differences in processivity, and we suspected that the single-stranded RNA (ssRNA) length of the primer (i.e., the number of bases that are not bound by the template) could play a role in RP synthesis as well.

To investigate this further, we performed a transcription reaction with a primer ending in 3′ UG (UG primer). Up to the point of realignment, this primer is extended into the same $\frac{UGCA}{UCGU}$ primer-template duplex as that of the 3′ AU primer on the wild-type template. However, because the UG primer has only 9 ssRNA bases after annealing to 3′ 2C of the promoter (compared to 10 ssRNA bases for the AU primer after annealing with 3′ 1U), we would expect an effect on RP formation. Indeed, during extension of the UG primer, equal levels of GP and RP were formed (Fig. 3B). Moreover, RP formation was abolished by mutation of 1U (Fig. 3B), without a change in the GP signal level, confirming the identity of the RP band. This thus demonstrates that IAV transcription processivity is affected by both the stability of the primer-template duplex and the ssRNA length of the primer.

### The priming loop suppresses realignment during transcription.

**FIG 4:**
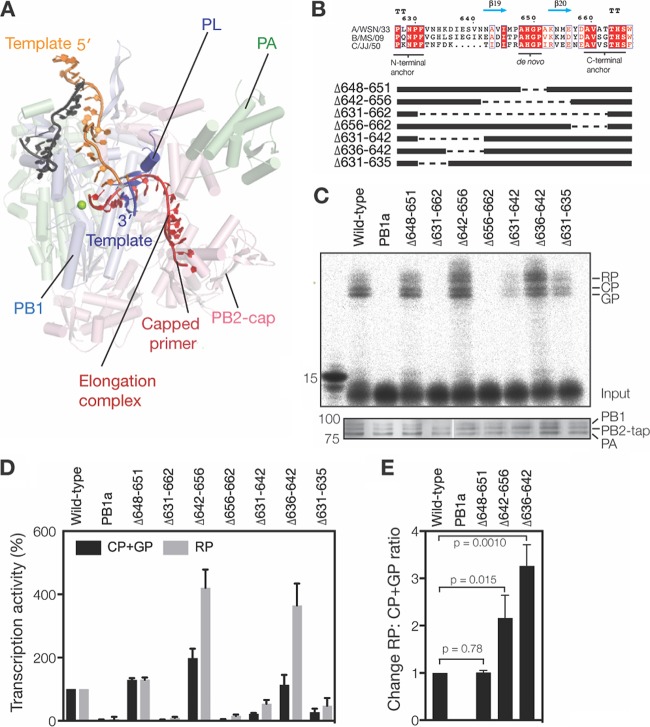
The priming loop affects transcription elongation. (A) Superposed structures of the influenza B virus RdRp (PDB accession number 5MSG) with the poliovirus 3D^pol^ RdRp elongation complex (PDB accession number 3OL7). For the poliovirus 3D^pol^ complex, part of the nascent strand (red) and 2 nucleotides of the template (dark blue) are shown. These superposed structures illustrate the putative path of the partially resolved capped primer (red) from the structure under PDB accession number 5MSG and the position of the priming loop (PL) relative to the putative path of the rest of the capped primer. (B) Amino acid alignment of the PB1 priming loop sequences of the influenza A/WSN/33 (H1N1), influenza B/Michigan/22687/09, and influenza C/JJ/50 viruses. Identical residues are shaded red, conserved residues are boxed in blue, and the secondary-structure interpretations are based on the structure under PDB accession number 4WSB. A schematic of the PB1 priming loop truncation mutants (7) used in this study is also shown. (C) Extension of a radiolabeled, capped, 11-nucleotide primer ending in 3′ AG in the presence of wild-type or mutant IAV RdRp purified from HEK 293T cells. The bottom panel shows SDS-PAGE analysis followed by silver staining of purified IAV RdRps. (D) Mean accumulation of transcription products initiated from 3G and 2C (GP+CP) or produced after realignment (RP). Error bars show standard deviations (*n* = 3). (E) Fold change in realignment relative to the wild type after normalization to the total transcription activity. Error bars show standard deviations (*n* = 3). The *P* values were determined by using an unpaired *t* test.

The above-described observations suggest that a component of the RdRp interacts with the primer to suppress realignment and increase processivity. Superposing the influenza B virus RdRp with a bound primer onto the poliovirus 3D^pol^ elongation complex shows that the PB1 priming loop, which is located between the active site and the entrance of the nascent strand exit channel, is ideally positioned to interact with the incoming capped primer (Fig. 4A). The PB1 priming loop was previously found to be crucial for viral replication initiation and prime realignment (6, 7). Moreover, it was shown previously that a deletion of residues 648 to 651 of the priming loop (Δ648–651) increases IAV transcription (6), which suggests that the priming loop may play a role in transcription initiation or elongation. To test whether the priming loop affects IAV transcription elongation, we purified a set of seven priming loop truncation mutants alongside active-site and wild-type controls (Fig. 4B and C), as described previously (7), and incubated them with the AG primer and NTPs. We observed clear GP and CP signals in six of the nine reactions as well as a double-RP band, likely because both GP and CP initiation products had been realigned (see also Fig. 3A). Measurement of the synthesized CP and GP signals showed that mutant Δ648–651 and a mutant lacking PB1 residues 642 to 656 (Δ642–656) extended the capped primer more efficiently than did the wild-type enzyme (Fig. 4C and D). This suggests that in the wild-type enzyme, the tip and β-sheet of the priming loop affect the elongation of the capped RNA primer. In contrast, mutant Δ636–642 synthesized CP and GP at levels that were indistinguishable from those of the wild type, while the four other priming loop mutants showed impaired primer extension levels (Fig. 4C and D), likely because they had a general activity impairment (7).

We next analyzed the effect of the priming loop on transcription elongation in more detail and measured the RP signal produced by the mutants. This analysis showed that transcriptional realignment appeared to be increased in reaction mixtures containing mutants Δ642–656 and Δ636–642 (Fig. 4C to E). After correction for the differences in the transcriptional activities, we found that the realignment efficiency of mutants Δ642–656 and Δ636–642, expressed as the change in the ratio of RP to CP plus GP, was significantly increased compared to that of the wild type, whereas mutant Δ648–651 was indistinguishable from the wild type (Fig. 4E). These results imply that the β-strand of the priming loop suppresses realignment during transcription elongation and thus plays a role in the processivity of IAV transcription. Due to the absence of sequence conservation in the β-strand of the loop (Fig. 4B), future crystallographic studies will be required to identify the mechanism for the interaction between the primer and the priming loop.

## DISCUSSION

**FIG 5:**
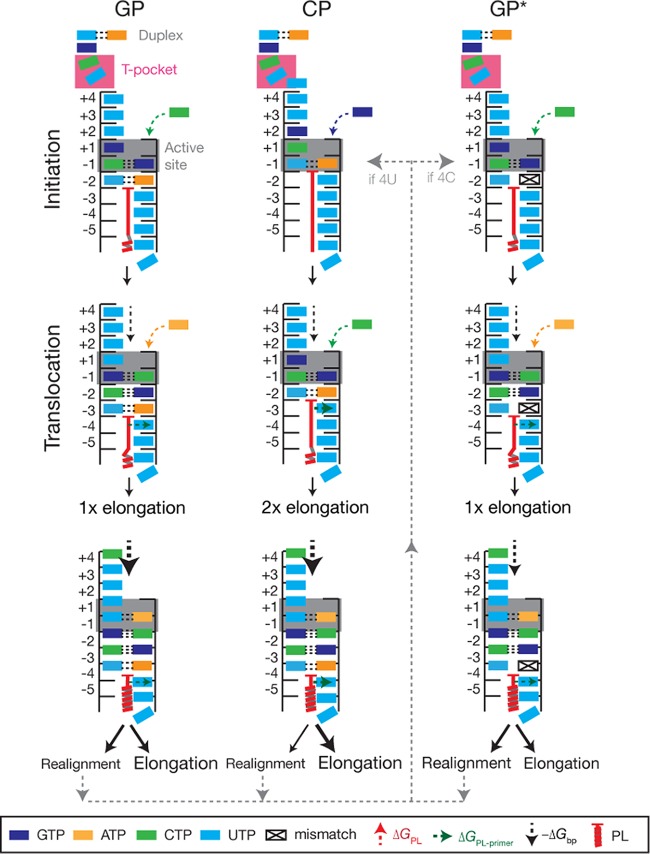
Model for influenza A virus transcription initiation and elongation. IAV transcription can initiate with a capped primer that fully base pairs with 3′ 1U and 2C of the vRNA (GP formation), a primer that base pairs with only 3′ 1U (CP formation), or a primer that Watson-Crick base pairs with only 2C and not 3′ 1U (GP* formation [non-Watson-Crick base pairing is indicated as a mismatch]). Based on the structure under PDB accession number 5MSG, 6 bases of the 3′ vRNA terminus are located in the template entry channel during GP synthesis, while three bases (3′ 7U, 8C, and 9G) remain single stranded between the duplex at the surface of the RdRp and the template entry channel. Of these three residues, 7U and 8C are stacked in a T-orientation by residues of the PB2 subunit (the T-pocket). It is likely that the interaction between PB2 and 7U is sequence specific, as a 7U→A mutation was previously shown to abrogate *de novo* initiation (30). The priming loop stabilizes the capped primer in the active site (Δ*G*_PL-primer_) to suppress realignment. When the 3′ 1U of the vRNA leaves position −4 of the active site, realignment may occur, depending on the stability of the template-primer duplex. On a vRNA promoter containing 3′ 4U, realignment will proceed (gray dotted line) via the CP pathway. On a vRNA promoter containing a 3′ 4C (segments 1, 2, and 3 of the IAV genome), realignment will proceed via the GP* pathway. It is known that this mutation downregulates transcription (25).

The mechanism of influenza virus transcription relies on the binding and cleavage of capped host cell mRNAs and the alignment of the cleavage products to the vRNA template (Fig. 1A). Cleavage is mediated by the PA endonuclease, which preferentially cleaves 3′ of G moieties *in vitro* (11, 12). A crystal structure of the influenza B virus RdRp bound to a vRNA template and a capped primer showed that the 3′ terminus of the vRNA can enter and even overshoot the active site by 1 nt without duplex unwinding (13). This places 2C and 3G in positions −1 and +1 of the active site (Fig. 5), which is ideal for transcription initiation with primers ending in 3′ G. Here we find that primers ending in 3′ G are indeed preferentially base paired with 2C of the template to allow initiation from 3G. However, a substantial fraction of the primers ending in G is also base paired with 1U, enabling initiation from 2C and suggesting that although Watson-Crick base pairing is important for initiation, it is not essential. Indeed, we also observe that U-U base pairs support efficient initiation, although A-A base pairs do not, which suggests that transcription initiation is subject to constraints in addition to base pairing, such as the shape of the primer-template helix.

When no G is available in the first 14 nt of the capped host mRNA, PA-mediated cleavage can occur at other bases *in vitro* (11, 12) and *in vivo* (15, 17). We find that primers ending in 3′ A or U are preferentially aligned to 1U of the template and extended from 2C (Fig. 2 and 3). We did not analyze transcription initiation from primers ending in 3′ C in this study. To support transcription initiation from 2C, residues 1U and 2C of the vRNA 3′ terminus must be placed at positions −1 and +1 of the active site, respectively (Fig. 5). If initiation from 3G is the default for the RdRp (13), initiation from 2C can occur only if the 3′ terminus backtracks 1 nt. This step would leave 5 nt in the template entry channel and 1 unpaired base upstream of the entry channel (Fig. 5). Although it is presently unknown where this unpaired base can be harbored in the structure, it is tempting to speculate that it can be accommodated below bases 7U and 8C, which are stacked in a T-orientation by residues of the PB2 subunit (the “T-pocket”). Given that we observed significant initiation from 2C with primers ending in G (Fig. 1C), backtracking of the template is likely a frequent event. An alternative explanation is that initiation from 2C is the default initiation position and that the template must track forward to facilitate initiation from 3G, but this notion is not supported by crystal structures at present.

Previous analyses of IAV mRNAs from infected cells identified GCA duplications in the 5′ termini of these mRNAs (15, 18). Our *in vitro* analysis of IAV transcription elongation suggests that these 3-nt duplications are the result of a realignment event that shifts a partially extended capped primer from residue 4U of the vRNA 3′ end to 1U (Fig. 2F and 5). It is unlikely that the duplications are generated through the cleavage of (partly) finished IAV transcription products and their subsequent reextension into full-length transcription products. First, we observed that the formation of the GCA duplications is dependent on template residues 1U and 4U (Fig. 2E and F) and not on 2C (Fig. 3A). The latter base allows the incorporation/presence of a G in the transcript, and this would be the preferred site for PA endonuclease cleavage (11, 12) over cleavage downstream of the A residue in the GCA sequence. Second, there is no clear evidence that cleavage products accumulate in reactions that rely on internal radioactive labeling, such as globin-primed transcription reactions (Fig. 1D). Finally, an extension-cleavage-extension reaction is likely more complicated than the proposed realignment mechanism, because it would require the removal of the partially extended primer from the product exit channel, a conformational change of the PB2 cap-binding domain to allow primer egress, cleavage of the transcript by the PA endonuclease, and reinsertion of the primer. Overall, we thus conclude that a realignment mechanism is the most parsimonious explanation for the findings reported here and in the literature.

In the wild-type RdRp, realignment events appear to be relatively rare (∼10% of elongation events) when the RdRp is extending a primer that can form a double-Watson-Crick base pair with the template (i.e., the AG primer). However, 15 other nucleotide combinations exist for the last 2 bases of the primer, which implies that realignment may be more frequent in viral infections. Indeed, in viral infections, up to 30% of transcription elongation events involve realignment, as shown by deep sequencing (15). Although we do not understand the importance of the realignment mechanism in IAV RNA synthesis, we observe an increase in the number of realignment events when (i) the primer and template are not aligned through Watson-Crick base pairing and (ii) the part of the primer that does not base pair with the template is relatively short (Fig. 3). Without the realignment mechanism, transcription initiation in the above-mentioned two scenarios would produce significantly fewer full-length IAV mRNAs (on a UG primer, ∼50% of elongation products are realigned) (Fig. 3B), which would limit viral protein synthesis in turn. We therefore speculate that the realignment mechanism is a means to rescue low-processive transcription events and limit abortive transcription initiation. However, the virus may also use this mechanism to regulate the expression of its genes. Indeed, segments with 3′ 4C in the vRNA promoter instead of the canonical 3′ 4U show little realignment (Fig. 2G), which may explain why transcription is downregulated on these IAV genome segments (25).

In summary, here we propose that the priming loop of the IAV RdRp interacts with the primer that it snatched from the host cell to improve transcription elongation, which, as we argue above, may be crucial for IAV mRNA and protein synthesis. This thus provides the IAV RdRp with two mechanisms to optimize viral transcription and help the enzyme elongate efficiently after a cap-snatching event. These observations provide a deeper insight into IAV RNA synthesis, and they may be relevant for other negative-strand RNA viruses that have been observed to use realignment mechanisms as well.

## MATERIALS AND METHODS

### Cells and plasmids.

Human embryonic kidney (HEK) 293T cells were maintained in Dulbecco's modified Eagle's medium (DMEM) (Sigma) supplemented with 10% fetal calf serum (FCS). Sf9 cells were grown in Xpress medium (Lonza) supplemented with penicillin and streptomycin. Plasmids pPolI-NA, pcDNA-NP, pcDNA-PB1, pcDNA-PA, pcDNA-PB2-TAP, and pcDNA-PB1a were described previously (20, 21, 26). Priming loop mutant PB1 Δ648–651 (6); PA endonuclease mutant D108A (27); and priming loop mutants Δ642–656, Δ636–642, Δ631–662, Δ656–662, Δ631–642, and Δ631–635 (7) were also described previously.

### Sequence alignment and structural modeling.

Amino acid sequences of the PB1 subunits of influenza A/WSN/33 (H1N1), influenza B/Michigan/22687/09, and influenza C/JJ/50 viruses were aligned by using ClustalX (28). The alignment was visualized by using ESPript (29). To superpose the poliovirus 3D^pol^ elongation complex (PDB accession number 3OL7) and the influenza B virus RdRp crystal structure (PDB accession number 5MSG), we aligned active-site residues 324 to 332 of the poliovirus enzyme with residues 442 to 449 of the bat influenza virus PB1 subunit in PyMOL 1.3.

### Capping and labeling RNA primers.

Synthetic 5′ tri- or diphosphate-containing RNAs of 11 nt (Table 1) (Chemgenes) were capped with a radiolabeled cap-1 structure by using 0.25 μM [α-^32^P]GTP (3,000 Ci mmol^−1^; Perkin-Elmer), 2.5 U/μl 2′-*O*-methyltransferase (NEB), and a vaccinia virus capping kit (NEB). The products were denatured in formamide and purified as described previously (6).

**TABLE 1:** RNA primers

Primer	Length (nt)	Sequence (5′–3′)
AG	11	ppGAAUACUCAAG
CA	11	pppGAAUACUCACA
UG	11	pppGAAUACUCAUG
AU	11	pppGAAUACUCAAU
AA	11	pppGAAUACUCAAA
vRNA 5′	15	AGUAGAAACAAGGCC
vRNA 3′	14	GGCCUGCUUUUGCU

### *In vitro* capped oligonucleotide extension assay.

Capped RNA oligonucleotide extensions were typically set up in 25-μl reaction mixtures containing 1 mM dithiothreitol (DTT), 5 mM MgCl_2_, 1 U/μl RNasin (Promega), 1,500 cpm capped RNA primer, 0.7 μM vRNA promoter (Sigma), 5% glycerol, 0.05% NP-40, 75 mM NaCl, 10 mM HEPES (pH 7.5), and ∼1 μM RdRp. The reaction mixtures were preincubated for 20 min at 30°C, and the reactions were then started by adding 500 μM UTP, 500 μM ATP, 500 μM CTP, and 500 μM GTP to the mixtures. Aliquots were taken at the time points indicated, and reactions were stopped with 4 μl formamide loading buffer. Samples were subsequently denatured and analyzed by 20% denaturing PAGE. The extended capped primers were visualized by phosphorimaging. *P* values were determined by using an unpaired *t* test. To sequence the extended capped primers, products were excised from the 20% denaturing PAGE gel, eluted overnight in water and desalted over NAP-10 columns (GE Healthcare). The isolated RNA was next polyadenylated with poly(A) polymerase (NEB), reverse transcribed with the dTGG primer (5′-CACGACGCTCTTCCGATCTTTTTTTTTTTTTTTTTTGG-3′), and turned into double-stranded DNA (dsDNA) by using second-strand primer 2ND_GA (5′-GTTCAGACGTGTGCTCTTCCGATCTGA+AT+A+CTCAAG-3′ [here “+” indicates a locked nucleic acid (LNA) base]). To remove the excess primer, the DNA was treated with exonuclease VII (NEB) for 1 h and subsequently heated to 95°C for 10 min to denature the exonuclease. Finally, the DNA was amplified with GoTaq (Promega) by using primers P5 (5′-AATGATACGGCGACCACCGAGATCTACACTCTTTCCCTACACGACGCTCTTCCGATCT-3′) and i7003 (5′-CAAGCAGAAGACGGCATACGAGATACTGGTGTGACTGGAGTTCAGACGTGTGCTCTTCCGATCT-3′) and TOPO cloned (Invitrogen) for Sanger sequencing. The β-globin mRNA transcription assay was performed as described previously (6).
